# Clinical Factors Associated with Abnormal Postures in Parkinson's Disease

**DOI:** 10.1371/journal.pone.0073547

**Published:** 2013-09-19

**Authors:** Tomoko Oeda, Atsushi Umemura, Satoshi Tomita, Ryutaro Hayashi, Masayuki Kohsaka, Hideyuki Sawada

**Affiliations:** Clinical Research Center and Department of Neurology, National Hospital of Utano, National Hospital Organization, Kyoto City, Kyoto, Japan; Oslo University Hospital, Norway

## Abstract

**Background:**

Abnormal posture (AP) is often seen in Parkinson's disease (PD), and marked forms known as dropped head syndrome and camptocormia encumber daily living activities. Unlike other motor disabilities such as bradykinesia or muscular rigidity, AP is not always improved but rather deteriorated by PD medication.

**Purpose:**

To clarify factors associated with neck and thoracolumbar AP.

**Methods:**

Neck flexion (NF) and thoracolumbar (TL) angles were measured in 216 consecutive PD patients and 175 elderly healthy controls. The differences in NF and TL angles between PD patients and controls were designated as ΔNFA and ΔTLA, respectively. The association of ΔNFA or ΔTLA and predictable factors such as age, sex, duration of PD, Hoehn Yahr (H–Y) stage, Unified Parkinson's Disease Rating Scale Part 3 (UPDRS-3), daily dose of dopamine agonists, and comorbid orthopedic spinal lesions was investigated in PD patients. Patients were divided into quartiles according to ΔNFA or ΔTLA. The association between predictable factors and ΔNFA or ΔTLA was estimated as odds ratio (OR), comparing with the lowest quartile as the reference by multivariate regression analysis.

**Results:**

Compared with controls, distributions of all three posture angles were significantly shifted rightward in PD patients. Although there were no difference in UPDRS-3 scores in the quartiles of ΔNFA, the highest quartile was associated with H–Y stage ≥3 [OR 2.99, 95% confidence interval (CI) 1.33–6.70, p = 0.008] after adjustment for age, sex and comorbid orthopedic spinal lesions. The highest quartile of ΔTLA was associated with comorbid orthopedic spinal lesions [OR 5.83 (1.42–23.8), p = 0.014], and UPDRS-3 score [OR 3.04 (1.80–5.15)/10 points, p<0.0001].

**Conclusion:**

Thoraco-lumbar AP was associated with UPDRS-3 scores and orthopedic spinal lesions, and in contrast, neck AP was not associated with these factors, suggesting that they had different pathomechanisms.

## Introduction

Abnormal posture (AP) is a frequent complication of Parkinson's disease (PD), and severe AP such as dropped head syndrome and camptocormia (marked bending of thoracolumbar spine) encumber the activities of daily living. To explore the pathogenesis of AP, the following issues should be addressed: disease specificity, definition, coexistence of two or more types of AP, and relationship to other extrapyramidal motor disturbances of PD. Bending posture, including a marked form called bent spine syndrome, is seen in elderly people without PD, suggesting that AP could occur in people without PD [1]. Therefore, comparison of body angles between PD patients and elderly healthy people is required. A consistent definition of AP including dropped head syndrome or camptocormia has not been established because the distribution of body angles has not been studied, and cut-off points between normal postures and AP cannot be set. Although various types of AP, anterocollis (dropped head), scoliosis, and camptocormia have been reported in patients with PD, it is unclear whether they are related to each other. A combination of two of more APs, such as dropped head and camptocormia, can be observed simultaneously; however, it has not been elucidated whether they are caused by the same pathological process or occur coincidentally. AP is thought to be an extrapyramidal sign because it can appear in untreated patients, as described in the historic paper by James Parkinson [2], and many clinical factors regarding severity of PD have been identified as risk factors for camptocormia [3], [4], [5], [6]. However, recent case reports indicate the association of dopaminergic replacement therapy, especially by dopamine agonists, and anterocollis [7], [8], [9], [10], [11], [12] or Pisa syndrome [13], [14], [15], [16], suggesting a drug-related phenomenon. Camptocormia is thought to be caused by muscular rigidity [17], [18] or axial dystonia [5], [19], [20], at least in the early stages of PD. In addition, myopathy [21], [22] and myositis [23] in the relevant muscles are also thought to be involved in AP. As described above, the precise mechanisms of AP have not been elucidated, and identification of factors that are associated with body angles is required to explore the pathogenesis of AP.

In the present study, three body angles, neck flexion (NF), fore-bent (FB) and lateral-bent (LB) angles were surveyed in PD patients and in healthy controls. The distribution of the body angles was compared between PD patients and controls. Clinical characteristics were investigated by the quartiles of body angle difference from the mean angle in the controls. The association of predictable variables was investigated using multinomial regression models. With reference to the lowest quartile of body angles, odds ratios (ORs) of predictable variables were estimated using multivariate multinomial regression.

## Materials and Methods

### Participants

From March to July 2010, we enrolled 221 consecutive patients with PD who were able to keep a standing position without any assistance, and were treated at the Center for Parkinson's Disease and Related Disorders, Utano National Hospital, Kyoto, Japan. A diagnosis of PD was made according to Steps 1 and 2 of the United Kingdom Parkinson's Disease Society Brain Bank Clinical Diagnostic Criteria [24]. AP can be seen in multiple system atrophy (MSA) [25] or progressive supranuclear palsy (PSP) [26]; therefore, patients with the possibility of MSA or PSP were excluded. Patients with possible or probable MSA according to the second consensus statement on MSA diagnosis [27] were excluded, and in addition, 1.5 Tesla brain magnetic resonance imaging (MRI) was performed in all individuals to rule out the possibility of MSA. Those with atrophy or signal changes in the putamen or middle cerebellar peduncles, atrophy of the cerebellum, or cross sign of the pons were excluded. Patients with possible or probable PSP according to the clinical criteria of the National Institute for Neurological Diseases and Stroke/Society for Progressive Supranuclear Palsy [28] were excluded, and those with atrophy of the midbrain tegmentum were excluded using brain MRI. Patients who had undergone deep brain stimulation were excluded because it could be related to AP [29], [30].

As healthy elderly controls, we recruited people registered with the Employment Service Center for Elderly People in Kyoto (a public registration center for small jobs or volunteers in the community) and spouses of PD patients who gave informed consent for the study. Posture angles were measured using the same procedures as for the patients.

The study was approved by the National Hospital Organization Utano Hospital Review Board, and all eligible individuals were informed of the purpose and methods of the study, and all of them provided informed consent. According to the comment from the committee, written consent was not required but oral consent was obtained because of the observational nature of the study and the photographs were not recognizable. The study investigators documented the oral consent of the participants in their medical records, and the committee approved the procedure to document oral consent.

### Measurement of NF, FB and LB angles

NF, FB and LB angles were measured on photographs of the lateral and back views of the participants in an upright position (in an “ON” period if the participants had motor fluctuations). On lateral view photographs, the angle between the two crossing lines—the line connecting the external acoustic foramen and the acromion, and the line connecting the acromion and the grater trochanter—was defined as the NF angle. Similarly, FB angle was the angle between the line connecting the acromion and the greater trochanter and a vertical line. On back view photographs, the angle between the line connecting the posterior process of the seventh cervical vertebra and that of the fifth lumbar vertebra and a vertical line was defined as the LB angle (Figure S1). NF and FB angles toward flexion and extension were expressed as positive and negative values, respectively, and LB angle was an absolute value because bending toward left or right sides occurred by chance.

### Data collection

Clinical data including age, sex, duration of PD, PD onset age, initial symptoms (tremor-dominant or not), Hoehn and Yahr (H–Y) stage, history of psychosis, and history of agonist-related AP were collected as possible predictors of AP. History of agonist-related AP was defined when AP occurred after dose escalation of dopamine agonists and recovered after discontinuation or de-escalation. In addition, scores on the Unified Parkinson's Disease Rating Scale Part 3 (UPDRS-3) were collected. Comorbid orthopedic spinal lesions (compression fracture of the vertebrae, disc hernia, or spondylolisthesis of the vertebrae) were diagnosed on plain X-ray or MRI performed in all participants within 6 months before the acquisition of body angle photographs. Information about regular physical exercise prior to entry was extracted from clinical records.

Daily doses of dopamine agonists and use of selegiline and amantadine at entry were also recorded. L-Dopa dose was calculated using the following formula: 1.0× regular levodopa dose or 1.25×regular levodopa dose if taking entacapone. The dose of dopamine agonists was calculated as the levodopa equivalent dose (LDED) according to the formula: LDED (mg)  =  pramipexole (mg) ×67 + ropinirole (mg) ×25 + pergolide (μg) ×67 + cabergoline (mg) ×67 + bromocriptine (mg) ×10 + talipexole (mg) ×67.

### Definition of body angle difference between PD and controls

The differences in NF angle (ΔNFA) and TL angle (ΔTLA) between PD patients and healthy controls were evaluated according to the following formulae:
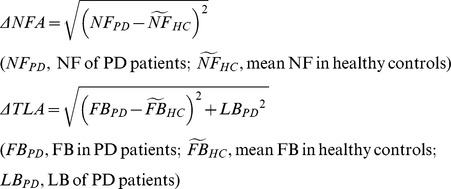



In addition FB angle (ΔFBA) was also investigated because there may be quite different mechanisms in lateral bending and antero-posterior bending APs.
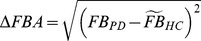



### Variables

Age, duration, onset age, daily dose of L-Dopa, dopamine agonists, and UPDRS-3 scores were regarded as scale variables. Sex, initial symptoms, H–Y stage (1–2 *vs.* 3–4), history of psychosis, history of agonist-related AP, orthopedic spinal lesions, and use/non-use of selegiline and amantadine were regarded as dichotomous variables.

### Statistical analysis

NF, FB and LB angles were compared between patients with PD and healthy elderly controls by Mann–Whitney *U* test because of non-Gaussian distributions. ΔNFA and ΔTLA were divided into quartiles. The association of predictable variables and ΔNFA or ΔTLA was statistically analyzed using multivariate multinomial regression models. The association was estimated as OR, compared with the lowest quartile as the reference. After adjustment for age, sex, and presence of orthopedic spinal lesions, suitable predictable variables were selected by forward stepwise likelihood test. A value of p<0.05 was considered statistically significant. Statistical analyses were performed using the statistical software program IBM SPSS Statistics version 19.0. Results are presented as mean (standard deviation; SD).

## Results

### PD patients and elderly healthy controls

As controls, 91 men and 84 women aged 70.0 (7.1) and 69.7 (8.2) years, respectively, gave informed consent and their body angles were measured. In the controls, NF angle was 21.1° (10.6) in men and 11.8° (10.5) in women, and FB angle was –5.5° (4.0) in men and –3.8° (3.3) in women. There were significant differences in NF and FB angles between male and female controls (p<0.001 and p = 0.005). Due to significant sex differences in the angles in the controls, ΔNFA, ΔTLA and ΔFBA were calculated after separation by sex.

Among 221 enrolled patients with PD, five were eliminated because of deep brain stimulation (n = 2) and missing data (n = 3) and the remaining 216 patients were analyzed. In 216 PD patients (83 men and 133 women) with a mean age of 69.0 (9.0) years, PD duration was 8.0 (4.9) years, and onset age was 61.0 (9.7) years. Ninety-nine patients (45.8%) had tremor-dominant onset, and 116 (53.7%) were H–Y 3 or 4 and UPDRS-3 score was 20.6 (10.8). Orthopedic spinal lesions on X-ray or MRI were found in 41 (19.0%) patients. Sixty-one patients (28.2%) experienced psychosis and 22 (10.2%) had a history of agonist-related AP. Mean daily dose of L-Dopa and dopamine agonists (LDED) was 392.7 (187) mg and 103.7 (135.3) mg, respectively. Mean NF, FB and LB angle was 22.9° (14.9), 3.9° (10.0) and 3.2° (3.5) degrees, respectively.

### Distributions of body angles in PD patients and controls

**Figure 1:**
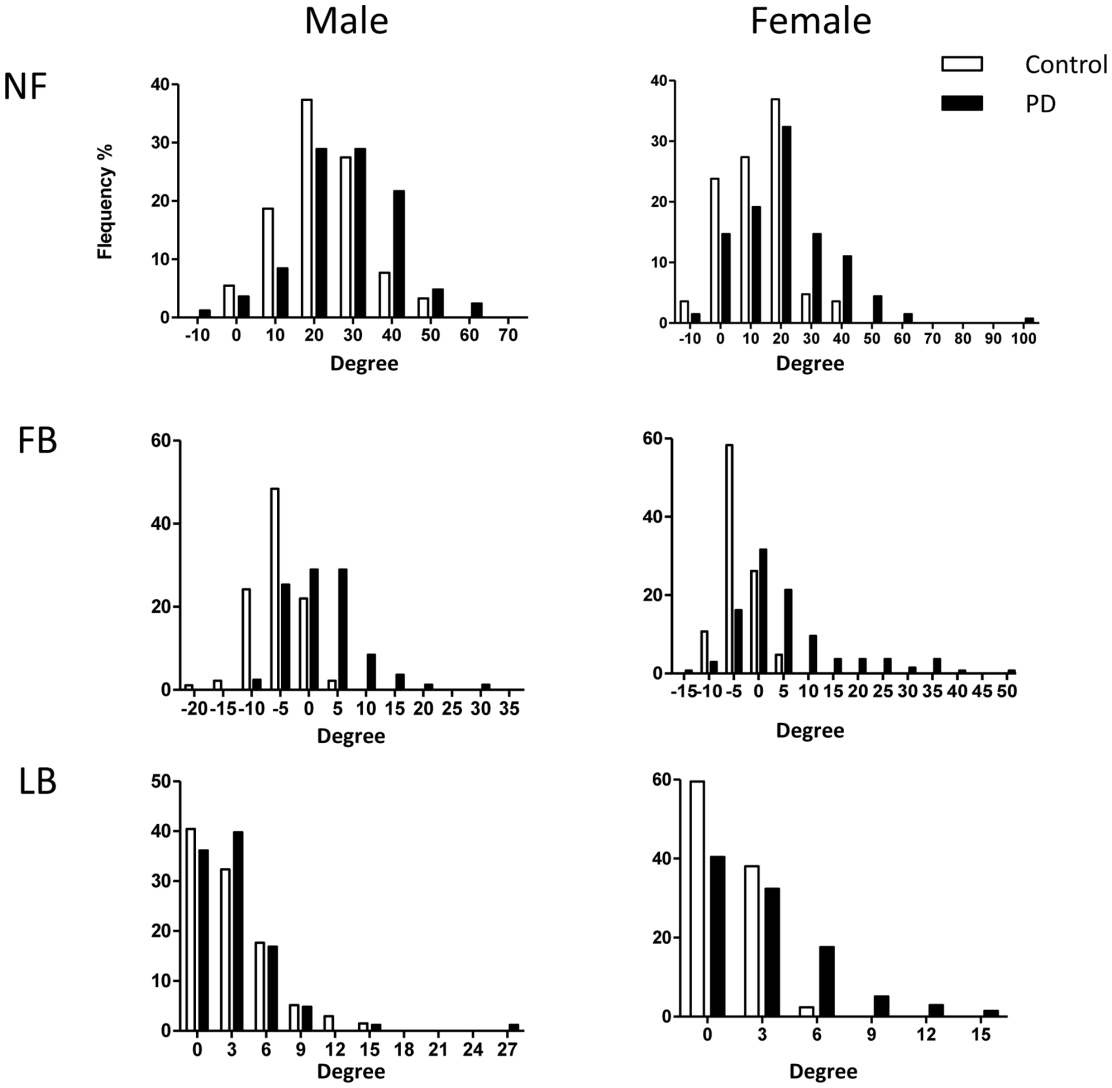
Surveyed posture angles in healthy controls (n = 175) and patients with PD (n = 216). NF angle and FB angle were distributed in a bell shape. They were shifted rightward in PD patients compared with healthy controls.

Distribution of NF, FB and LB angles in patients with PD and healthy controls is shown by sex in Figure 1. The distribution shifted rightward in the PD patients compared with the controls. All three posture angles in PD patients were significantly larger than in the controls in men and women (male NF, p = 0.0003; male FB, p<0.0001; male LB, p<0.0001; female NF, p = 0.0001; female FB, p<0.0001; female LB, p<0.0001). It was difficult to establish a cut-off point for AP because of the continuous distribution of the body angles in patients with PD.

### Factors associated with body angles in PD

There was no correlation between ΔNFA and ΔTLA (Pearson's correlation 0.066, p = 0.336). The demographic data by the quartiles of ΔNFA or ΔTLA are shown in Table 1 and Table 2. As for NFA (Table 1), H–Y and history of agonist-related AP differed significantly between the quartiles. Interestingly mean UPDRS-3 scores were very similar in the quartiles of ΔNFA as shown in Table 1, suggesting that severity of neck flexion AP were independent of overall motor disability.

**Table 1:** Demographic data by quartiles of neck-flexion angle.

		1st quartile				2nd quartile				3rd quartile				4th quartile				
		n = 60				n = 50				n = 50				n = 56				
		(0.1–4.9)				(5.1–9.9)				(10.2–16.9)				(17.2–83.2)				*p*
Age (Y), mean (SEM)		69.4	(	1.1	)	68.1	(	1.4	)	67.8	(	1.3	)	70.3	(	1.1	)	0.45
Sex, n (%)	Female Male	40 20	( (	66.7% 33.3%	) )	25 25	( (	50.0% 50.0%	) )	32 18	( (	64.0% 36.0%	) )	36 20	( (	64.3% 35.7%	) )	0.285
Duration (Y), mean (SEM)		7.7	(	0.7	)	7.2	(	0.7	)	8.3	(	0.6	)	8.6	(	0.6	)	0.44
Onset age(Y)		61.8	(	1.3	)	60.9	(	1.4	)	59.5	(	1.5	)	61.7	(	1.2	)	0.61
Initial symptoms	Tremor dominant Others	25 35	( (	41.7% 58.3%	) )	25 25	( (	50.0% 50.0%	) )	22 28	( (	44.0% 56.0%	) )	27 29	( (	48.2% 51.8%	) )	0.81
H-Y	1, 2	32	(	53.3%	)	30	(	60.0%	)	22	(	44.0%	)	16	(	28.6%	)	0.007
	3, 4	28	(	46.7%	)	20	(	40.0%	)	28	(	56.0%	)	40	(	71.4%	)	
UPDRS-3, mean (SEM)		20.3	(	1.6	)	21.1	(	1.6	)	20.7	(	1.6	)	20.4	(	1.0	)	0.98
Orthopedic spinal lesions	Yes No	12 48	( (	20.0% 80.0%	))	9 41	( (	18.0% 82.0%	) )	8 42	( (	16.0% 84.0%	) )	12 44	( (	21.4% 78.6%	) )	0.90
History of psychosis	Yes No	18 42	( (	30.0% 70.0%	) )	10 40	( (	20.0% 80.0%	) )	14 36	( (	28.0% 72.0%	) )	19 37	( (	33.9% 66.1%	) )	0.45
Agonist-related history	Yes No	9 51	( (	15.0% 85.0%	) )	2 48	( (	4.0% 96.0%	) )	2 48	( (	4.0% 96.0%	) )	9 47	( (	16.1% 83.9%	) )	0.0498
Dopa, mean (SEM)	mg/day	384	(	27.1	)	375	(	27	)	402	(	27	)	411	(	22	)	0.75
Agonist LDED, mean (SEM)	mg/day	104	(	20	)	105	(	18	)	111	(	21	)	96	(	15	)	0.95
Dopa + Agonist, mean (SEM)	mg/day	488	(	32	)	480	(	31	)	512	(	35	)	506	(	26	)	0.87
Selegiline Use	Use No use	23 37	( (	38.3% 61.7%	) )	23 27	( (	46.0% 54.0%	) )	20 30	( (	40.0% 60.0%	) )	29 27	( (	51.8% 48.2%	) )	0.46
Amantadine Use	Use No use	19 41	( (	31.7% 68.3%	) )	7 43	( (	14.0% 86.0%	) )	12 38	( (	24.0% 76.0%	) )	15 41	( (	26.8% 73.2%	) )	0.19
Rehabilitation	Yes No	9 51	( (	15.0% 85.0%	) )	6 44	( (	12.0% 88.0%	) )	8 42	( (	16.0% 84.0%	) )	11 45	( (	19.6% 80.4%	) )	0.75

*p* was calculated by ANOVA(scale variables) or by Pearson Chi square test (categorical variables).

**Table 2:** Demographic data by quartiles of thoracolumbar angle.

		1st quartile				2nd quartile				3rd quartile				4th quartile				
		n = 56				n = 51				n = 52				n = 57				
		(0.2–3.9)				(4.0–7.1)				(7.4–11.2)				(11.5–54)				*p*
Age (Y), mean (SEM)		66.7	(	1.2	)	66.6	(	1.5	)	71.1	(	1.2	)	71.4	(	1.0	)	0.003
Sex, n (%)	Female Male	35 21	( (	62.5% 37.5%	) )	30 21	( (	58.8% 41.2%	) )	29 23	( (	55.8% 44.2%	) )	39 18	( (	68.4% 31.6%	) )	0.561
Duration (Y), mean (SEM)		6.9	(	0.5	)	7.1	(	0.6	)	8.2	(	0.6	)	9.5	(	0.8	)	0.021
Onset age(Y)		59.8	(	1.3	)	59.5	(	1.4	)	62.9	(	1.4	)	61.9	(	1.1	)	0.21
Initial symptoms	Tremor dominant Others	29 27	( (	51.8% 48.2%	) )	24 27	( (	47.1% 52.9%	) )	25 27	( (	48.1% 51.9%	) )	21 36	( (	36.8% 63.2%	) )	0.42
H-Y	1, 2 3, 4	32 24	( (	57.1% 42.9%	) )	31 20	( (	60.8% 39.2%	) )	23 29	( (	44.2% 55.8%	) )	14 43	( (	24.6% 75.4%	) )	0.0005
UPDRS-3, mean (SEM)		16.2	(	1.2	)	18.7	(	1.4	)	19.7	(	1.1	)	27.4	(	1.7	)	<0.0001
Orthopedic spinal lesions	Yes No	3 53	( (	5.4% 94.6%	) )	7 44	( (	13.7% 86.3%	) )	13 39	( (	25.0% 75.0%	) )	18 39	( (	31.6% 68.4%	) )	0.002
History of psychosis	Yes No	10 46	( (	17.9% 82.1%	) )	11 40	( (	21.6% 78.4%	) )	13 39	( (	25.0% 75.0%	) )	27 30	( (	47.4% 52.6%	) )	0.002
Agonist-related history	Yes No	2 54	( (	3.6% 96.4%	) )	3 48	( (	5.9% 94.1%	) )	9 43	( (	17.3% 82.7%	) )	8 49	( (	14.0% 86.0%	) )	0.06
Dopa, mean (SEM)	mg/day	340	(	25	)	337	(	26	)	456	(	23	)	437	(	25	)	0.0003
Agonist LDED, mean (SEM)	mg/day	115	(	18	)	136	(	23	)	120	(	20	)	49	(	8	)	0.003
Dopa + Agonist, mean (SEM)	mg/day	454	(	32	)	473	(	37	)	576	(	28	)	486	(	25	)	0.029
Selegiline Use	Use No use	23 33	( (	41.1% 58.9%	))	25 26	( (	49.0% 51.0%	) )	25 27	( (	48.1% 51.9%	) )	22 35	( (	38.6% 61.4%	) )	0.63
Amantadine Use	Use No use	8 48	( (	14.3% 85.7%	) )	11 40	( (	21.6% 78.4%	) )	18 34	( (	34.6% 65.4%	) )	16 41	( (	28.1% 71.9%	) )	0.08
Rehabilitation	Yes No	8 48	( (	14.3% 85.7%	) )	4 47	( (	7.8% 92.2%	) )	8 44	( (	15.4% 84.6%	) )	14 43	( (	24.6% 75.4%	) )	0.12

*p* was calculated by ANOVA(scale variables) or by Pearson Chi square test (categorical variables).

In Table 2, age, disease duration, H–Y, UPDRS-3, orthopedic spinal lesions, history of psychosis and daily dose of L-Dopa and dopamine agonists were differed significantly between the quartile of ΔTLA (Table 2). These data were very similar in analysis of ΔFBA (Table S1).

There was strong multicollinearity between age and PD onset age; therefore, PD onset age was excluded. L-Dopa+agonist dose was eliminated from predictable variables because there was multicollinearity between L-Dopa dose and L-Dopa+agonist dose, (Figure S2). All the other predictive variables were incorporated into the multivariate multinomial regression models. The model showed that the highest quartile ΔNFA was significantly associated with H–Y 3 or 4 and the ORs were 2.99 [95% confidence interval (CI) 1.33–6.70, p = 0.008] with reference to the lowest quartile (Table 3). In contrast to ΔNFA, the highest quartile ΔTLA was associated with orthopedic spinal lesions [OR 5.83 (1.42–23.8), p = 0.014], and UPDRS-3 score [OR 3.04 (1.80–5.15)/10 points, p<0.0001] (Table 4). These data were similar in analysis of ΔFBA, but highest quartile was negatively associated with daily dose of agonists (Table S2).

**Table 3:** Odds ratios of factors for quartiles of neck flexion angle.

		2nd quartile							3rd quartile							4th quartile						
Predictable variables		OR (95% CI)						*p*	OR (95% CI)						*p*	OR (95% CI)						*p*
Age	/Year	0.88	(	0.56	–	1.38	)	0.57	0.77	(	0.49	–	1.22	)	0.27	0.97	(	0.61	–	1.53	)	0.89
Sex	Male Female (Ref)	2.01 1	(	0.92	–	4.35	)	0.08	1.08 1	(	0.49	–	2.39	)	0.85	1.07 1	(	0.49	–	2.34	)	0.87
Orthopedic spinal lesions	Yes No (Ref)	1.07 1	(	0.38	–	2.98	)	0.90	0.77 1	(	0.27	–	2.18	)	0.62	0.84 1	(	0.32	–	2.20	)	0.72
Hoehn-Yahr	III, IV I, II (Ref)	0.79 1	(	0.35	–	1.78	)	0.57	1.73 1	(	0.78	–	3.88	)	0.18	2.99	(	1.33	–	6.7	)	0.008
																1						
The reference category is: 1st quartile.																						

predictable variables: forced entry (age, sex, and orthopedic lesions), forward stepwise likelihood ratio test (duration of PD, H-Y, history of psychosis, history of agonist-related AP, UPDRS-3, Dopa dose, DA agonist dose, amantadine use, selegiline use, and rehabilitation).

**Table 4:** Odds ratios of factors for quartiles of thoracolumbar angle.

		2nd quartile							3rd quartile							4th quartile						
Predictable variables		OR (95% CI)						*p*	OR (95% CI)						*p*	OR (95% CI)						*p*
Age	/Year	0.94	(	0.59	–	1.49	)	0.79	1.58	(	0.94	–	2.66	)	0.08	1.01	(	0.60	–	1.71	)	0.96
Sex	Female	0.94	(	0.41	–	2.18	)	0.892	1.09	(	0.46	–	2.57	)	0.84	1.78	(	0.72	–	4.40	)	0.21
	Male (Ref)	1							1							1						
Orthopedic spinal lesions	Yes	3.41	(	0.78	–	14.8	)	0.102	3.58	(	0.89	–	14.4	)	0.073	5.83	(	1.42	–	23.8	)	0.014
	No (Ref)	1							1							1						
UPDRS–3	/10 points	1.65	(	0.99	–	2.77	)	0.06	1.40	(	0.84	–	2.34	)	0.20	3.04	(	1.80	–	5.15	)	<0.0001
Dopa daily dose	/100mg	0.91	(	0.72	–	1.15	)	0.44	1.33	(	1.03	–	1.71	)	0.03	1.07	(	0.83	–	1.39	)	0.59
Agonist daily dose	/100mg LDED	1.21	(	0.90	–	1.63	)	0.21	1.20	(	0.88	–	1.63	)	0.25	0.64	(	0.39	-	1.07	)	0.09
The reference category is: 1st quartile.																						

predictable variables: forced entry (age, sex, and orthopedic lesions), forward stepwise likelihood ratio test (duration of PD, H-Y, history of psychosis, history of agonist-related AP, UPDRS-3, Dopa dose, DA agonist dose, amantadine use, selegiline use, and rehabilitation).

## Discussion

All of the NF, FB and LB angles in PD were significantly larger than those in healthy controls. Distributions of NF and FB angles were bell-shaped both in PD patients and controls, and were shifted to the right in PD patients (Figure 1). Although distribution of LB angle was not bell-shaped because of absolute values, it was also shifted rightward in patients with PD. These data demonstrate that there is no distinct threshold of body angles for AP, but rather suggest the possibility that the truncal body angles are shifted in most PD patients. Although extremely abnormal postures such as dropped head syndrome or camptocormia are rare, AP could be a common problem in PD. These observations suggest that right-shifted body angle in PD is related to extrapyramidal symptoms such as axial rigidity or dystonia, which is reflected in impaired perception of body angles [31], [32], [33].

Previous studies have identified many clinical factors as risk factors for camptocormia, such as duration of PD [3], age [3], H–Y stage [3], [4], UPDRS-3 [3], [4], [5], axial rigidity [6], levodopa daily dose [4], levodopa treatment duration [3], dementia [3], autonomic dysfunction [4], and history of vertebral surgery [3]. The present study revealed that ΔTLA was associated with comorbid orthopedic spinal lesions and UPDRS-3 score. In contrast to ΔNFA, ΔTLA was associated with UPDRS-3 scores after adjustment comorbid orthopedic spine lesions, suggesting that thoracolumbar AP might be a part of motor disturbance of PD.

There are no reports of risk of neck AP such as anterocollis or dropped head in PD. In the present study, ΔNFA was associated only with H–Y stage ≥3. As shown in Table 1 there were no difference in UPDRS-3 scores between quartiles of ΔNFA. These data suggest that neck AP would be associated with postural reflex disturbance but not with overall motor disturbance of PD and patients with neck flexion AP may have relatively mild motor symptoms in limbs compared to axial symptoms including postural reflex disturbance.

Unexpectedly, age did not contribute significantly to AP. Spinal lesions were significantly associated with thoracolumbar AP, and this finding was consistent with a series of previous reports about camptocormia [3], [6], [34]. Although osteoporosis is a common complication of postmenopausal women [35], [36] and PD patients [37], the significant association with spinal lesions was revealed to be independent of sex, age and motor symptom severity in the present study. Severe spinal lesions seem to have some direct effect on vertebral alignment; however, another possible pathomechanism can be raised, namely, spinal lesions may trigger a peripheral-trauma-induced dystonia [38], [39] in paraspinal muscles. It has been reported that peripheral trauma induces movement disorders, and dystonia is the most frequent type (72%) [40]. PD may become a predisposing factor for trauma-induced dystonia because dystonia is one of the frequent complications in patients with PD. Additionally, subsequent secondary muscle or soft tissue degeneration might fix truncal AP in patients with advanced PD.

Although several antiparkinsonian drugs such as dopamine agonists[7], [8], [9], [10], [16], monoamine oxidase B inhibitor [14] (only selegiline is available in Japan to date), amantadine [12] or entacapone/levodopa/carbidopa [15] are nominated for causes of AP, they were not significant risk factors in our study.

Possible association of other factors such as contaminant myopathy or disturbance of body angle sensation could not be assessed in the present study, and these points are limitations of the study. Although this study had another limitation owing to its cross-sectional observational nature, the results suggest that body angles are shifted rightward in most patients with PD, and it is difficult to establish a clear cut-off point for body angles. TL AP is independent of NF AP and the significant risk factor for TL AP was comorbid orthopedic spinal lesions and motor disturbance of PD.

## Supporting Information

Figure S1 Schematic representation of body angles. NF angle was defined as the angle between the two crossing lines: a line connecting the external acoustic foramen and the acromion, and another line connecting the acromion and the greater trochanter. Similarly, FB angle was the angle between a line connecting the acromion and the greater trochanter and a vertical line. On back view photographs, the angle between the line connecting the posterior process of the seventh cervical vertebra and that of the fifth lumbar vertebra and a vertical line was defined as LB angle.
(TIF)

Figure S2 Scattered plots of scale predictable variables. The relationship between possible predictable variables (age, onset age, duration, UPDRS-3, and daily dose of L-Dopa, dopamine agonists and dose of Dopa+agonists) was investigated in scattered diagram. There was a linear correlation between age and onset age, and therefore PD onset age was excluded from statistical analysis. There was multicollinearity between L-Dopa dose and L-Dopa+agonist dose, and therefore L-Dopa+agonist dose was eliminated from predictable variables.
(TIF)

Table S1 (DOCX)

Table S2 (DOCX)
