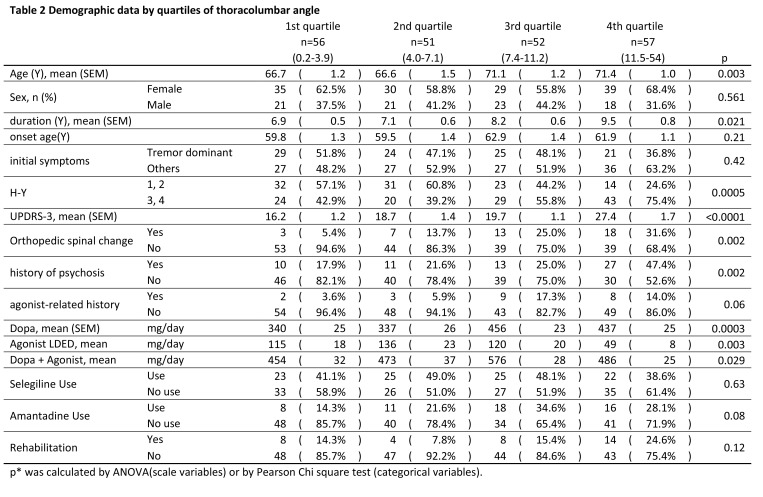# Correction: Clinical Factors Associated with Abnormal Postures in Parkinson's Disease

**DOI:** 10.1371/annotation/d5228e50-2098-44c9-b663-829dd9b4b97e

**Published:** 2013-12-13

**Authors:** Tomoko Oeda, Atsushi Umemura, Satoshi Tomita, Ryutaro Hayashi, Masayuki Kohsaka, Hideyuki Sawada

Several errors were introduced in the formatting of Tables 1 and 2 during the preparation of this article for publication. Please find the correct versions of each table here:

Table 1: 

**Figure:**
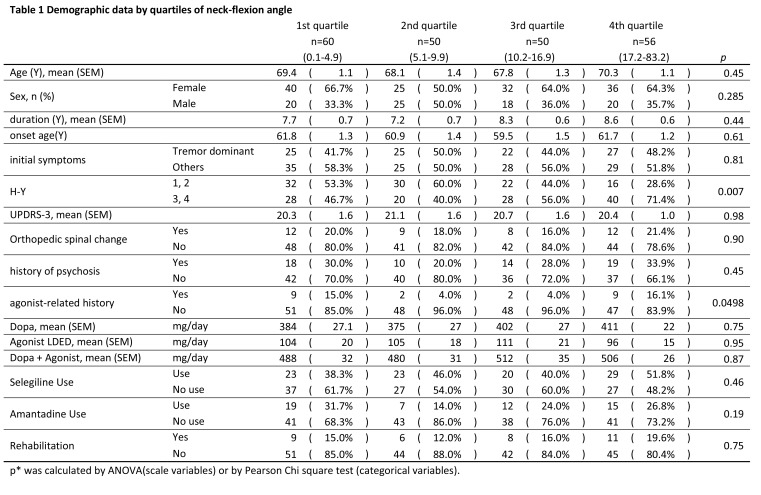


Table 2: 

**Figure:**