# Supplementary figures and images for: Clinical Factors Associated with Abnormal Postures in Parkinson's Disease
Source: PLoS One. 2013 Sep 19;8(9):e73547. doi: 10.1371/journal.pone.0073547 (PMC3777935; doi:10.1371/journal.pone.0073547)

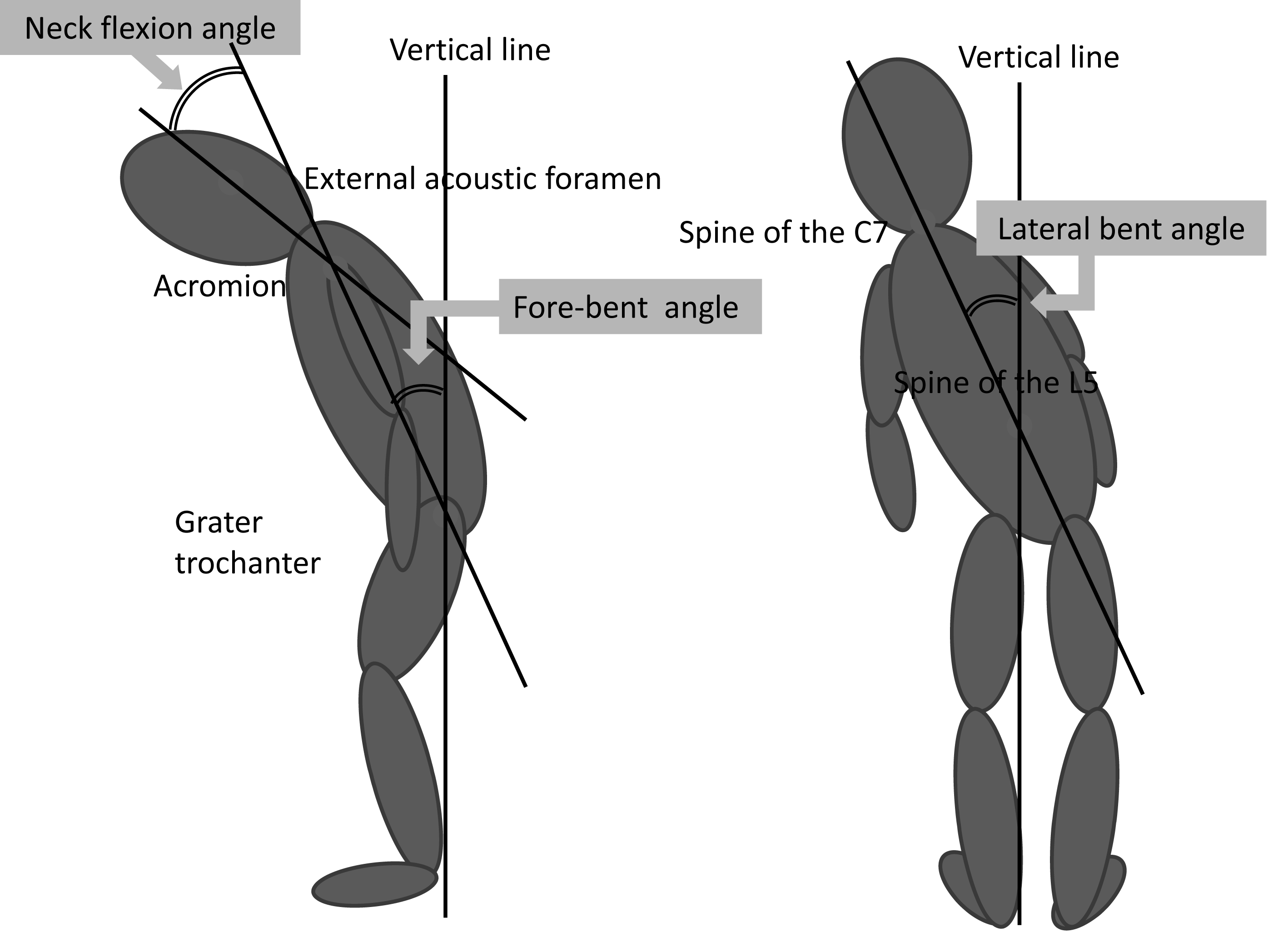

Supplement: Figure S1 — Schematic representation of body angles. NF angle was defined as the angle between the two crossing lines: a line connecting the external acoustic foramen and the acromion, and another line connecting the acromion and the greater trochanter. Similarly, FB angle was the angle between a line connecting the acromion and the greater trochanter and a vertical line. On back view photographs, the angle between the line connecting the posterior process of the seventh cervical vertebra and that of the fifth lumbar vertebra and a vertical line was defined as LB angle. (TIF) [file pone.0073547.s001.tif]

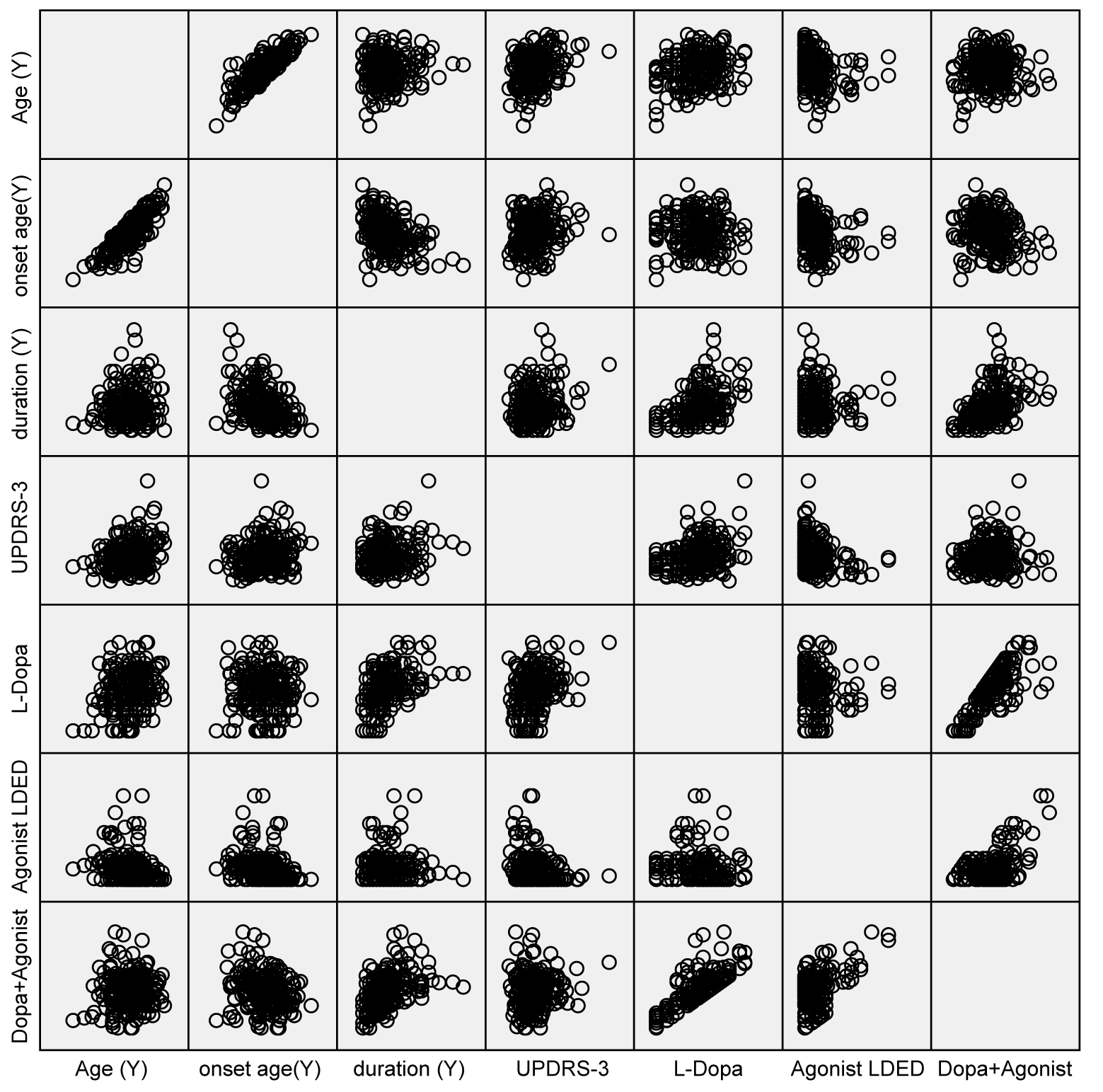

Supplement: Figure S2 — Scattered plots of scale predictable variables. The relationship between possible predictable variables (age, onset age, duration, UPDRS-3, and daily dose of L-Dopa, dopamine agonists and dose of Dopa+agonists) was investigated in scattered diagram. There was a linear correlation between age and onset age, and therefore PD onset age was excluded from statistical analysis. There was multicollinearity between L-Dopa dose and L-Dopa+agonist dose, and therefore L-Dopa+agonist dose was eliminated from predictable variables. (TIF) [file pone.0073547.s002.tif]
