# Supplementary material for: Clinical Factors Associated with Abnormal Postures in Parkinson's Disease
Source: PLoS One. 2013 Sep 19;8(9):e73547. doi: 10.1371/journal.pone.0073547 (PMC3777935; doi:10.1371/journal.pone.0073547)
Supplement: Table S1 — (DOCX) [file pone.0073547.s003.docx]

| **Table S1 Demographic data by quartiles of forebent angle** | | | | |  |  |  |  |  |  |  |  |  |  |  |  |  |  |
| --- | --- | --- | --- | --- | --- | --- | --- | --- | --- | --- | --- | --- | --- | --- | --- | --- | --- | --- |
|  | | 1st quartile | | | | 2nd quartile | | | | 3rd quartile | | | | 4th quartile | | |  |  |
|  |  | n=56 | | | | n=53 | | | | n=54 | | | | n=53 | | | |  |
|  |  | (0.2-3.2) | | | | (3.5-5.8) | | | | (6.5-10.8) | | |  | (11.5-54) | | | | *p* |
| Age (Y), mean (SEM) | | 67.3 | ( | 1.2 | ) | 67.2 | ( | 1.2 | ) | 69.7 | ( | 1.3 | ) | 71.8 | ( | 1.0 | ) | 0.020 |
| Sex, n (%) | Female | 35 | ( | 62.5% | ) | 30 | ( | 56.6% | ) | 32 | ( | 59.3% | ) | 36 | ( | 67.9% | ) | 0.660 |
|  | Male | 21 | ( | 37.5% | ) | 23 | ( | 43.4% | ) | 22 | ( | 40.7% | ) | 17 | ( | 32.1% | ) |  |
| Duration (Y), mean (SEM) | | 7.1 | ( | 0.6 | ) | 7.7 | ( | 0.6 | ) | 7.4 | ( | 0.6 | ) | 9.6 | ( | 0.9 | ) | 0.039 |
| Onset age(Y) | | 60.2 | ( | 1.4 | ) | 59.5 | ( | 1.3 | ) | 62.2 | ( | 1.4 | ) | 62.2 | ( | 1.2 | ) | 0.33 |
| Initial symptoms | Tremor dominant | 30 | ( | 53.6% | ) | 24 | ( | 45.3% | ) | 25 | ( | 46.3% | ) | 20 | ( | 37.7% | ) | 0.43 |
|  | Others | 26 | ( | 46.4% | ) | 29 | ( | 54.7% | ) | 29 | ( | 53.7% | ) | 33 | ( | 62.3% | ) |  |
| H-Y | 1, 2 | 37 | ( | 66.1% | ) | 24 | ( | 45.3% | ) | 28 | ( | 51.9% | ) | 11 | ( | 20.8% | ) | 0.00003 |
|  | 3, 4 | 19 | ( | 33.9% | ) | 29 | ( | 54.7% | ) | 26 | ( | 48.1% | ) | 42 | ( | 79.2% | ) |  |
| UPDRS-3, mean (SEM) |  | 16.2 | ( | 1.3 | ) | 19.3 | ( | 1.2 | ) | 18.9 | ( | 1.0 | ) | 28.3 | ( | 1.7 | ) | <0.0001 |
| Orthopedic spinal lesions | Yes | 3 | ( | 5.4% | ) | 7 | ( | 13.2% | ) | 14 | ( | 25.9% | ) | 17 | ( | 32.1% | ) | 0.001 |
|  | No | 53 | ( | 94.6% | ) | 46 | ( | 86.8% | ) | 40 | ( | 74.1% | ) | 36 | ( | 67.9% | ) |  |
| History of psychosis | Yes | 8 | ( | 14.3% | ) | 15 | ( | 28.3% | ) | 13 | ( | 24.1% | ) | 25 | ( | 47.2% | ) | 0.002 |
|  | No | 48 | ( | 85.7% | ) | 38 | ( | 71.7% | ) | 41 | ( | 75.9% | ) | 28 | ( | 52.8% | ) |  |
| Agonist-related history | Yes | 3 | ( | 5.4% | ) | 3 | ( | 5.7% | ) | 8 | ( | 14.8% | ) | 8 | ( | 15.1% | ) | 0.15 |
|  | No | 53 | ( | 94.6% | ) | 50 | ( | 94.3% | ) | 46 | ( | 85.2% | ) | 45 | ( | 84.9% | ) |  |
| Dopa, mean (SEM) | mg/day | 348 | ( | 23 | ) | 360 | ( | 29 | ) | 424 | ( | 22 | ) | 441 | ( | 26 | ) | 0.02 |
| Agonist LDED, mean (SEM) | mg/day | 131 | ( | 21 | ) | 128 | ( | 23 | ) | 109 | ( | 15 | ) | 44 | ( | 8 | ) | 0.002 |
| Dopa + Agonist, mean (SEM) | mg/day | 480 | ( | 33 | ) | 489 | ( | 37 | ) | 533 | ( | 27 | ) | 485 | ( | 25 | ) | 0.60 |
| Selegiline Use | Yes | 27 | ( | 48.2% | ) | 23 | ( | 43.4% | ) | 24 | ( | 44.4% | ) | 21 | ( | 39.6% | ) | 0.84 |
|  | No | 29 | ( | 51.8% | ) | 30 | ( | 56.6% | ) | 30 | ( | 55.6% | ) | 32 | ( | 60.4% | ) |  |
| Amantadine Use | Yes | 9 | ( | 16.1% | ) | 13 | ( | 24.5% | ) | 16 | ( | 29.6% | ) | 15 | ( | 28.3% | ) | 0.34 |
|  | No | 47 | ( | 83.9% | ) | 40 | ( | 75.5% | ) | 38 | ( | 70.4% | ) | 38 | ( | 71.7% | ) |  |
| Rehabilitation | Yes | 6 | ( | 10.7% | ) | 5 | ( | 9.4% | ) | 10 | ( | 18.5% | ) | 13 | ( | 24.5% | ) | 0.11 |
|  | No | 50 | ( | 89.3% | ) | 48 | ( | 90.6% | ) | 44 | ( | 81.5% | ) | 40 | ( | 75.5% | ) |  |
| *p* was calculated by ANOVA(scale variables) or by Pearson Chi square test (categorical variables). | | | | | | | | | | | | |  |  |  |  |  |  |
