# Supplementary material for: Clinical Factors Associated with Abnormal Postures in Parkinson's Disease
Source: PLoS One. 2013 Sep 19;8(9):e73547. doi: 10.1371/journal.pone.0073547 (PMC3777935; doi:10.1371/journal.pone.0073547)
Supplement: Table S2 — (DOCX) [file pone.0073547.s004.docx]

| **Table S2 Odds ratios of factors for quartiles of forebent angle** | | | | | | |  |  |  |  |  |  |  |  |  |  |  |  |  |  |  |  |  |  |
| --- | --- | --- | --- | --- | --- | --- | --- | --- | --- | --- | --- | --- | --- | --- | --- | --- | --- | --- | --- | --- | --- | --- | --- | --- |
|  |  | **2nd quartile** | | | | | | |  | **3rd quartile** | | | | | | |  | **4th quartile** | | | | | | |
| Predictable variables | | OR (95% CI) | | | | | | *p* |  | OR (95% CI) | | | | | | *p* |  | OR (95% CI) | | | | | | *p* |
| Age | /Year | 0.86 | ( | 0.55 | - | 1.36 | ) | 0.52 |  | 1.10 | ( | 0.68 | - | 1.75 | ) | 0.71 |  | 0.92 | ( | 0.54 | - | 1.57 | ) | 0.76 |
| Sex | Female | 0.91 | ( | 0.41 | - | 2.01 | ) | 0.807 |  | 0.92 | ( | 0.41 | - | 2.08 | ) | 0.85 |  | 1.64 | ( | 0.66 | - | 4.09 | ) | 0.29 |
|  | Male (Ref) | 1 |  |  |  |  |  |  |  | 1 |  |  |  |  |  |  |  | 1 |  |  |  |  |  |  |
| Orthopedic spine lesions | Yes | 2.81 | ( | 0.66 | - | 11.9 | ) | 0.16 |  | 5.63 | ( | 1.47 | - | 21.5 | ) | 0.012 |  | 6.22 | ( | 1.53 | - | 25.2 | ) | 0.011 |
|  | No (Ref) | 1 |  |  |  |  |  |  |  | 1 |  |  |  |  |  |  |  | 1 |  |  |  |  |  |  |
| UPDRS-3 | /10 points | 1.62 | ( | 0.98 | - | 2.66 | ) | 0.06 |  | 1.38 | ( | 0.84 | - | 2.28 | ) | 0.20 |  | 3.33 | ( | 1.95 | - | 5.66 | ) | <0.0001 |
| Agonist daily dose | /100mg LDED | 1.03 | ( | 0.79 | - | 1.36 | ) | 0.81 |  | 0.96 | ( | 0.72 | - | 1.28 | ) | 0.79 |  | 0.51 | ( | 0.30 | - | 0.88 | ) | 0.02 |
| The reference category is: 1st quartile. | | | | | | |  |  |  |  |  |  |  |  |  |  |  |  |  |  |  |  |  |  |
|  |  |  |  |  |  |  |  |  |  |  |  |  |  |  |  |  |  |  |  |  |  |  |  |  |
| predictable variables: forced entry (age, sex, and orthopedic lesions), forward stepwise likelihood ratio test (duration of PD, HY, history of psychosis, history of agonist-related AP, UPDRS-3, Dopa dose, DA agonist dose, amantadine use, selegiline use, and rehabilitation) | | | | | | | | | | | | | | | | | | | | | | | | |
|  |  |  |  |  |  |  |  |  |  |  |  |  |  |  |  |  |  |  |  |  |  |  |  |  |
|  |  |  |  |  |  |  |  |  |  |  |  |  |  |  |  |  |  |  |  |  |  |  |  |  |
